# The influence of psychosocial stress on functional connectivity and neuroendocrine markers in adolescents with depressive and comorbid anxiety disorders: a study protocol

**DOI:** 10.1186/s12888-025-07689-0

**Published:** 2025-12-10

**Authors:** Ricarda Jacob, Alexandra Otto, Irina Jarvers, Stephanie Kandsperger, Angelika Ecker, Daniel Schleicher, Wilhelm M. Malloni, Inga D. Neumann, Romuald Brunner

**Affiliations:** 1 Department of Child and Adolescent Psychiatry and Psychotherapy, University of Regensburg, Universitaetsstraße 84, 93053 Regensburg, Germany; 2 Department of Cognitive Neuroscience, University of Regensburg, Regensburg, Germany; 3 Department of Behavioural and Molecular Neurobiology, University of Regensburg, Regensburg, Germany

**Keywords:** Adolescence, Major depressive disorder, Anxiety disorders, Functional connectivity, Oxytocin, Cortisol, Alpha-amylase, Montreal imaging stress task, Psychosocial stress

## Abstract

**Background:**

Psychosocial stress is a major risk factor for adolescent depression and anxiety, impacting neurodevelopment through hypothalamic-pituitary-adrenal axis and oxytocinergic system dysregulation. Stress-related alterations in fronto-limbic resting-state functional connectivity (rsFC) have been linked to depression and anxiety disorders, yet the role of oxytocin in these processes remains unclear. Existing research often excludes comorbid anxiety or focuses on adults, limiting insights into developmental trajectories and stress-related brain changes in youth. This study aims to investigate the association of peripheral oxytocin, cortisol, and α-amylase and fronto-limbic rsFC in response to psychosocial stress in adolescents with depressive disorders, comparing those with and without comorbid anxiety disorders and healthy controls.

**Methods:**

This study will include a total of 90 participants (aged 12–17 years), comprising three groups (*n* = 30 per group): (1) adolescents with Major depressive disorder (MDD), (2) MDD with comorbid anxiety disorder, and (3) healthy controls. After a clinical examination and psychometric assessment, participants undergo Magnetic Resonance Imaging to assess rsFC before and after stress induction. Furthermore, Diffusion Tensor Imaging is conducted. Psychosocial stress is induced using the Montreal Imaging Stress Task, which requires participants to solve arithmetic tasks under time and social pressure. Saliva samples are collected at multiple time points to analyse oxytocin, cortisol and α-amylase levels.

**Discussion:**

This study offers valuable insights into the neurobiological mechanisms of stress in adolescents with depression and comorbid anxiety disorders. By examining the relationship between fronto-limbic rsFC and endocrine responses, the findings may inform the development of more targeted interventions, such as neuromodulation techniques, to improve treatment outcomes for this vulnerable population.

**Trial registration:**

This study is registered with the German Clinical Trials Register the date of registration was 28 August 2023 (registration number DRKS00032507).

## Introduction

### Theoretical background

Chronic or repeated psychosocial stress is a critical risk factor for the development and maintenance of depressive and anxiety disorders in children and adolescents [1]. These disorders often emerge during adolescence and are associated with long-term impairments in quality of life, increased risk of chronicity, and substantial societal costs (e.g., disability-adjusted life years) [2]. The developmental stage marks a key period of vulnerability, characterized by substantial remodelling of both functional and structural brain connectivity [3, 4]. Diffusion Tensor Imaging (DTI) studies highlighted ongoing development of white matter tracts involved in emotion regulation and cognitive control [5] with alterations in fronto-limbic connectivity associated with depressive and anxiety symptoms in youth [6, 7].

Additionally, stress exposure contributes to maladaptive trajectories of neurodevelopment, increasing the risk for depressive and anxiety disorders [8, 9]. Chronic stress has been linked to dysregulation of key stress-responsive systems, particularly the hypothalamic-pituitary-adrenal (HPA) axis [10] and the oxytocinergic system [11, 12], both critical for emotion regulation and social functioning [13, 14]. While gonadal hormones have been studied in relation to sex differences in adolescent brain maturation and depressive and anxiety disorders [15], the influence of neuropeptides like oxytocin (OXT) in stress-related neurodevelopmental alterations remains underexplored. Recent work by Bernhard et al. [16] underscores the complexity of the adolescent psychoneuroendocrine stress response in major depressive disorder (MDD), showing altered stress reactivity, including OXT pathways, in this population. These findings complement the broader understanding of how neuroendocrine dysregulation, including OXT and cortisol (CORT), may underlie vulnerability to depressive and anxiety disorders in adolescence [17] .

Neuroendocrine responses to acute stress influence large-scale brain networks [18], yet their effects on fronto-limbic resting-state functional connectivity (rsFC) in adolescents with depressive and anxiety disorders remain poorly understood. The fronto-limbic network—encompassing the amygdala, prefrontal cortex, and anterior cingulate cortex (ACC)—is central to stress adaptation and emotion regulation [19, 20]. Aberrant rsFC within this network has been linked to depressive and anxiety disorders, often reflecting hyperconnectivity in stress-sensitive regions and diminished top-down regulatory control [21, 22]. While studies have linked fronto-limbic rsFC to psychiatric symptoms, many have relied on cross-sectional designs or focused on adults [21], limiting insights into developmental trajectories and stress-related brain changes in youth. Moreover, comorbid anxiety is frequently excluded or assessed only dimensionally, constraining the ability to distinguish between depression alone and depression with comorbid anxiety [21]. Given the high rates of comorbidity [23] and the distinct clinical profiles of these subgroups, a more nuanced approach is necessary to understand how acute psychosocial stress affects fronto-limbic connectivity in adolescent populations.

Beyond connectivity patterns, the neuropeptide OXT has emerged as a potential modulator of fronto-limbic interactions, given its role in social cognition and affect modulation [24]. Peripheral OXT levels have been linked to individual differences in stress responsivity and social withdrawal, both common in youth with depressive and anxiety disorders [25]. While intranasal OXT has been shown to modulate rsFC within emotion regulation networks in healthy adults [26], it remains unknown whether endogenous, stress-induced OXT release similarly affects fronto-limbic rsFC. Furthermore, studies examining OXT’s role in stress-related connectivity changes have yielded inconsistent findings, likely due to differences in sampling methods (e.g., plasma vs. saliva), timing of measurements, and potential confounds like medication use [12].

In parallel, CORT—the end product of HPA axis activation—has been widely studied as a biomarker of stress-related psychopathology [27]. Evidence on OXT-CORT interactions is mixed: some human studies suggest that OXT buffers CORT reactivity [28, 29], while others report that higher stress-induced OXT is associated with increased CORT release and faster recovery [30]. Dysregulated post-stress CORT secretion, often observed in individuals with depressive and anxiety disorders [16, 31], has been associated with altered rsFC [32], particularly in circuits involved in emotional processing and self-regulation [33, 34]. In addition to CORT and OXT, salivary α-amylase (sAA), a marker of sympathetic nervous system activity [35], should be assessed simultaneously [36]. Altered sAA rhythms and reactivity have been observed in adolescents with depression and anxiety [37, 38], underscoring its relevance for understanding neuroendocrine dysregulation in these conditions.

However, little is known about how CORT interacts with OXT and sAA in shaping stress-induced rsFC changes. To our knowledge, no study has examined whether individual differences in OXT, CORT, and sAA reactivity predict fronto-limbic rsFC alterations following acute stress in adolescents with anxiety and depressive disorders.

### Study aim

This study aims to examine associations between peripheral OXT, sAA, and CORT release, and fronto-limbic rsFC in response to psychosocial stress in adolescents with depressive and anxiety disorders, compared to healthy controls. By explicitly comparing adolescents with depression alone, comorbid anxiety, and healthy controls, this research seeks to clarify neurobiological mechanisms underlying depressive and anxiety disorders during a critical period of neurodevelopment.

### Hypotheses

We expect blunted CORT and OXT stress responses in adolescents with MDD (with or without comorbid anxiety) relative to healthy controls, whereas baseline levels of these hormones are not expected to differ systematically across groups. For rsFC, we hypothesize time (pre- vs. post-stress) × group (MDD, COM, HC) interactions within fronto-limbic circuits, with greater alterations in adolescents with depressive disorders compared to healthy controls. Exploratorily, we expect that youth with MDD and comorbid anxiety will show more pronounced endocrine blunting and stronger fronto-limbic rsFC alterations than those with MDD alone. Finally, we hypothesize that stress-induced fronto-limbic rsFC changes will be significantly associated with endocrine reactivity (CORT, OXT, sAA), reflecting shared neuroendocrine mechanisms of stress adaptation.

## Methods

### Study design

This single-centre experimental study includes two experimental groups—adolescents diagnosed with depressive disorder and those with comorbid depressive and anxiety disorders—based on Diagnostic and Statistical Manual of Mental Disorders-IV (DSM-IV) and International Statistical Classification of Diseases and Related Health Problems-10 (ICD-10) criteria. Both groups are compared to a matched healthy control group. All participants undergo the same study procedure.

### Sample size and power analysis

An a-priori power analysis was carried out with G*Power 3.1 [39]. We modelled a one-way fixed-effects Analysis of Variance (ANOVA) with three independent groups (healthy controls [HC], major depressive disorder [MDD], and MDD with comorbid anxiety [COM]).

Previous rsFC studies in adolescents with MDD report medium to large effect sizes. Connolly et al. [40] demonstrated reduced amygdala–prefrontal rsFC in adolescents with MDD compared to controls (medium effect; *d* ≈ 0.4–0.6), although numerical effect sizes were not directly reported. Cullen et al. [41] reported group differences in amygdala–prefrontal connectivity that, when derived from published means and standard deviations, correspond to large effects (*d* ≈ 1.0–1.3).

Assuming α = 0.05 (two-tailed) and a desired power of 1-*β* = 0.80, the analysis indicated a required total sample size of *N* = 84 (28 per group). To account for potential data loss due to head motion or dropout, we plan to include *N* = 90 participants, i.e. 30 per group.

### Eligibility criteria

Eligibility is initially assessed via telephone screening and confirmed at the first study appointment. Participants must be adolescents aged 12–17 years. For the clinical groups, inclusion requires a primary diagnosis of at least moderate depression; the comorbid group additionally requires an anxiety disorder diagnosis. In case of discrepancy between clinical diagnoses and the results according to the structured clinical interview M.I.N.I.-KID, final group assignment is determined through expert consensus. Psychotropic medication and hormonal contraceptives are noted and will be corrected for but are not exclusion criteria. Full inclusion and exclusion criteria are listed in Table 1.

**Table 1:** Inclusion and exclusion criteria

**Inclusion Criteria**	**General:** • Adolescents aged 12–17 years • Sufficient understanding of the German language • Written informed consent from participant and legal guardian
	**Depressive Group**: Primary diagnosis of depressive disorders: • Moderate depressive episode (F32.1) • Severe depressive episode without psychotic symptoms (F32.2) • Severe depressive episode with psychotic symptoms (F32.3) • Major depressive disorder, recurrent, moderate (F33.1) • Major depressive disorder, recurrent, severe without psychotic symptoms (F33.2) • Depressive disorder, recurrent, severe with psychotic symptoms (F33.3)
	**Comorbid Group**: Primary diagnosis of depressive disorders and an additional anxiety disorder: • Agoraphobia with/without panic disorder (F40.00/F40.01) • Social phobia (F40.1) • Panic disorder (F41.0) • Generalized anxiety disorder (F41.1) • Separation anxiety disorder of childhood (F93.0) • Social anxiety disorder of childhood (F93.2) • Generalized anxiety disorder of childhood (F93.80)
	**Control Group**: Matched by sex and age; school type, handedness, pubertal status, contraceptive intake are controlled for
**Exclusion Criteria**	**General**: • Pregnancy/lactation • Pubertas praecox vera • Acute suicidal behavior • Glucocorticoid-containing medication • Genetic syndromes • Traumatic brain injury • Endocrinological or immune disorders • Chronic neurological disorders affecting brain development and physiology
	**Cognitive/Developmental**: • Intellectual impairment (IQ < 80) • Attendance at a school for special needs
	**MRI-Specific**: • Metallic foreign bodies (e.g., braces, cochlear implants, non-removable piercings) • Upper-body tattoos • Claustrophobia
	**Psychiatric Disorders**: Current/past diagnosis of: • Psychotic disorders • Autism • Bipolar disorder • Mental and behavioural disorders due to psychoactive substance use • Anorexia nervosa (if BMI < 16,5)
	**Control Group-Specific**: • Current or past psychiatric disorders • In-/outpatient psychiatric or psychotherapeutic treatment

### Recruitment

**Fig. 1:**
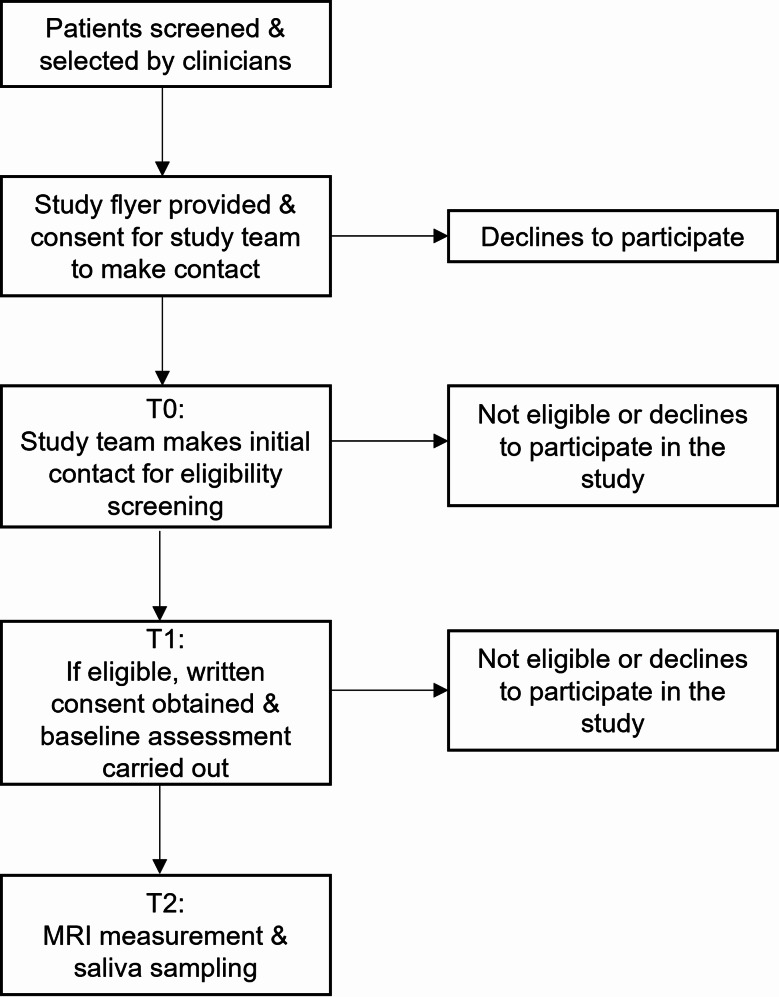
*Flow diagram.* MRI = Magnetic Resonance Imaging; T0 = telephone interview; T1 = first appointment; T2 = second appointment

Participants with at least moderate depressive disorder are recruited from the inpatient, outpatient, and day clinic units of the Clinic of Child and Adolescent Psychiatry, Psychosomatics, and Psychotherapy at the University of Regensburg, Germany. Healthy controls are contacted via the residents’ registration office and through announcements on mailing lists and the department’s website. Interested individuals complete an initial eligibility screening via telephone. Figure 1 outlines the study timeline and potential dropout points.

### Study procedure

**Fig. 2:**
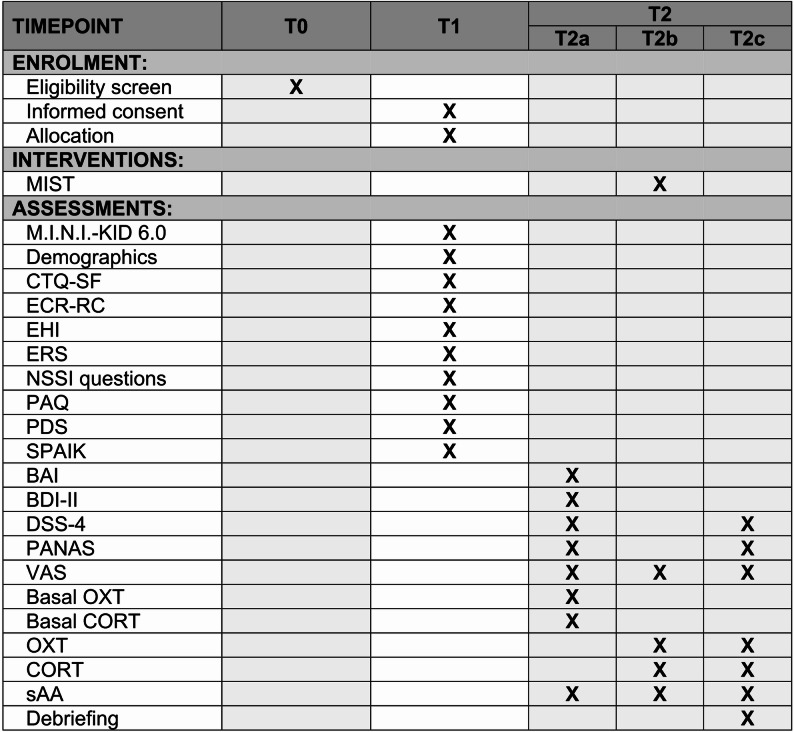
Study schedule. MIST = Montreal Imaging Stress Task; M.I.N.I.-KID 6.0 = Mini-International Neuropsychiatric Interview for Children and Adolescents; SPAIK = Social Phobia and Anxiety Inventory for Children; PAQ = Perth Alexithymia Questionnaire – German version; CTQ – SF = Child Trauma Questionnaire–Short Form; PDS = Pubertal Development Scale; NSSI = Non-Suicidal Self-Injury; ECR-RC = Experiences in Close Relationships Scale—Revised Child version; ERS = Emotion Reactivity Scale; EHI = Edinburgh Handedness Inventory; BAI = Beck Anxiety Inventory; BDI-II = Beck Depression Inventory-II; PANAS = Positive and Negative Affect Schedule; DSS-4 = Dissociation-Tension Scale; VAS = Visual Analogue Scale; OXT = Oxytocin, CORT = Cortisol; sAA = salivary α-amylase; T0 = telephone interview; T1 = first appointment; T2 = second appointment; T2a = Time previous to MIST, T2b = MIST, T2c = Time after MIST

Prior to inclusion, potential participants complete a telephone screening (T0) to assess eligibility. The study involves two appointments. During the first appointment (T1, ~ 1.5 h), participants and their legal guardian receive detailed study information. After providing written informed consent, participants complete the Mini-International Neuropsychiatric Interview for Children and Adolescents (M.I.N.I-KID 6.0) and a battery of questionnaires (see Fig. 2).

The second appointment (T2) involves Magnetic Resonance Imaging (MRI) measurements and saliva collection for the analysis of OXT, CORT and sAA. T2 lasts approximately 2.5 h and begins with a 20-minute relaxation period, during which participants complete questionnaires and engage in non-stressful activities such as colouring, reading, or listening to music. Participants then complete a practise run of the Montreal Imaging Stress Task (MIST) to determine task difficulty and ensure familiarity before the scan.

rsFC MRI scans are conducted before and after stress induction. Functional MRI (fMRI) is used to measure brain activity during both control and stress conditions, while DTI assesses structural brain connectivity. During rsFC scans, participants fixate on a central fixation cross; during DTI, age-appropriate cartoons are shown to maintain engagement.

**Fig. 3:**
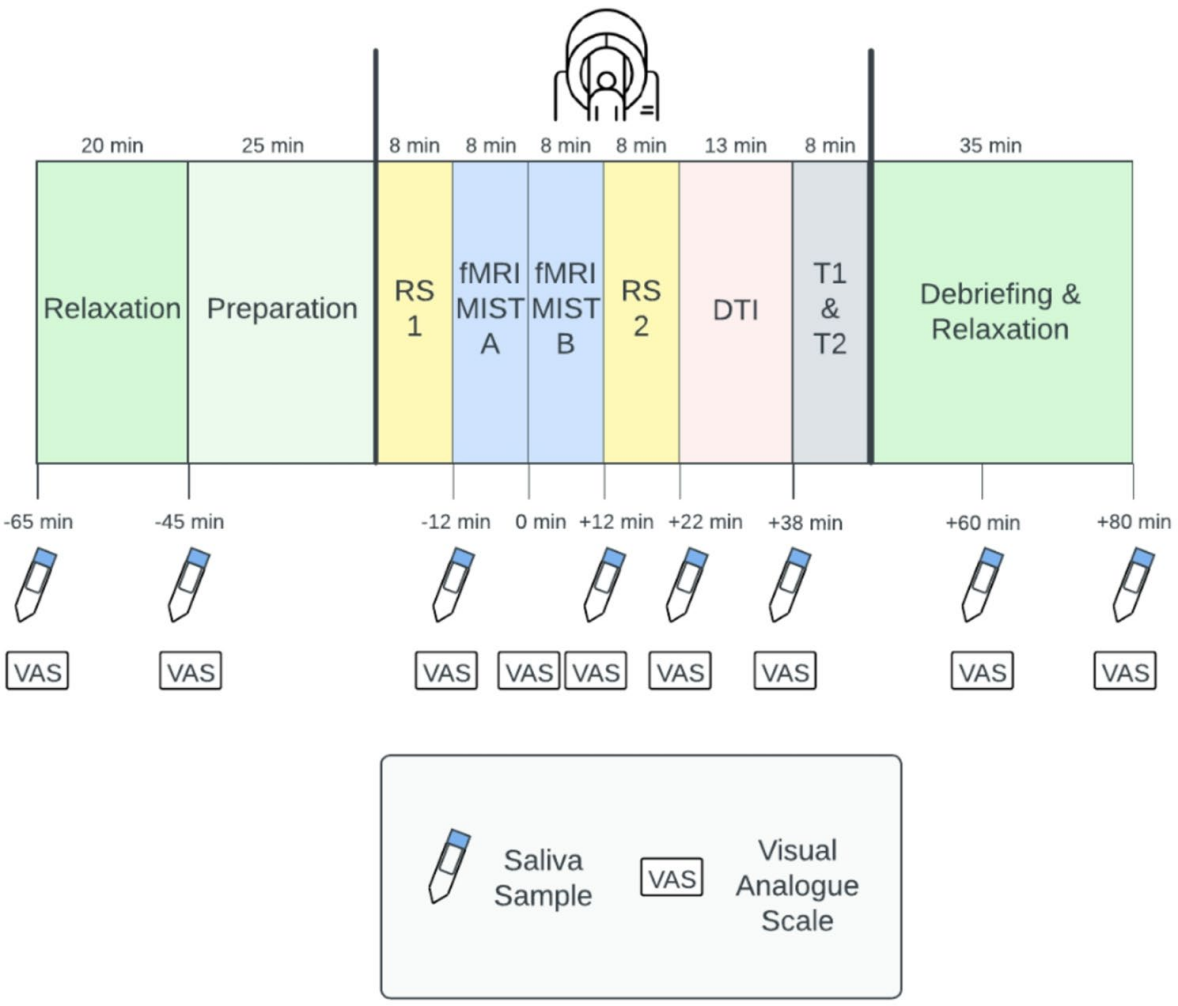
Illustration of the experimental procedure of T2. RS = Resting State; fMRI = functional Magnetic Resonance Imaging; MIST = Montreal Imaging Stress Task; DTI = Diffusion Tensor Imaging; T1 & T2 = T1 & T2-weighted images. Saliva Samples are collected for measurement of oxytocin, cortisol and α-amylase

Saliva samples for OXT, CORT, and sAA are collected at multiple time points before and after stress exposure (see Fig. 3). Psychological states, including stress, anxiety, anger, shame, and guilt, are assessed using Visual Analogue Scales (VAS).

### MIST paradigm

**Fig. 4:**
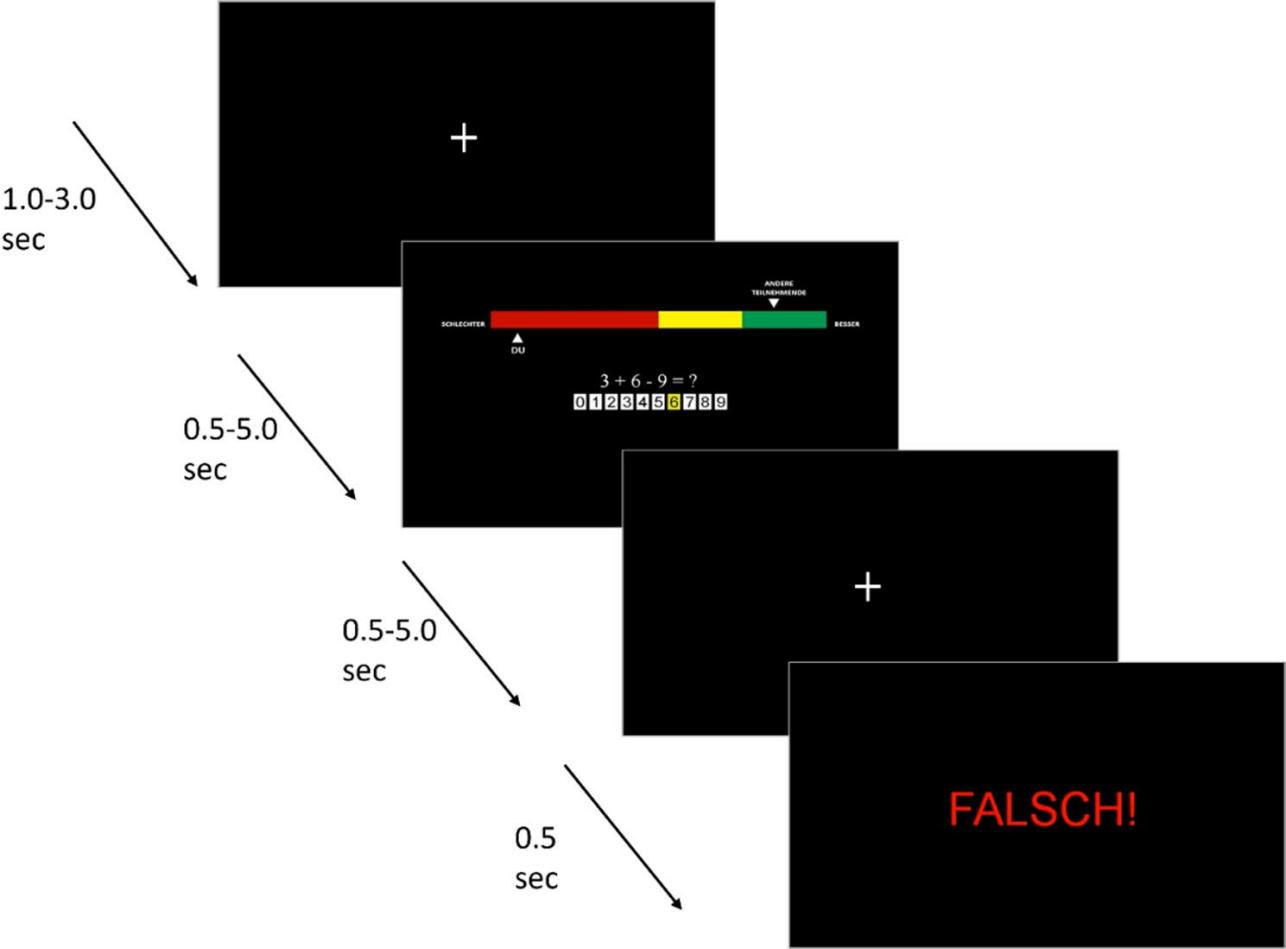
Trial structure of the MIST stress scan. “FALSCH” is the German word for “WRONG”

A modified version of the MIST, a demanding mental arithmetic task adapted for fMRI, is employed to induce psychosocial stress [42]. The paradigm follows a block-design with two consecutive 8-minute scans: a Control MIST followed by a Stress MIST, each consisting of 54 trials. Trials lasts 6 s and are followed by a 1–3 s inter-trial interval displaying a fixation cross. Each trial features a unique math problem with multiple-choice answers (0–9), a response window of 0.5–5 s and 0.5 s of visual feedback (“Right,” “Wrong,” or “Time out”). Each scan concludes with a 14-second fixation cross (see Fig. 4). The fixed order, Control followed by Stress MIST, was chosen as it avoids carryover effects and reduces variability, enhancing the assessment of individual differences in the stress response.

Before scanning, participants complete practice problems to familiarize themselves with the task and determine difficulty. Problems range from easy (e.g., single-digit addition/subtraction) to hard (e.g., operations involving multiple double-digit numbers). Each participant’s difficulty level, based on practise response times, remains constant across both scans.

Before the Control scan, reassuring statements like, “It’s okay if you don’t answer all the math problems correctly.” aim to reduce participants’ stress. During this scan, participants are given sufficient time to respond (about 5 s per problem) and receive prerecorded positive auditory feedback (e.g., “You are doing great! Looks like you’ve got that under control! Good job!”).

Before the Stress scan, participants are informed that high accuracy is required and that their data may be excluded if performance is insufficient. They are told that previous participants answered over 80% of the questions correctly and that their data will only be included if they match this level. During the scan, they receive prerecorded negative feedback (e.g., “You are not doing as well as we had hoped. Please do your best to answer these correctly”). A performance bar appears at the top of the screen, comparing the participant’s performance (usually in the red zone) to an apparent group average (in the green zone) (see Fig. 4). To reinforce a sense of failure, the response window is dynamically adjusted using a stair-step procedure: it decreases by 0.5 s after a correct answer and increases by 0.5 s after an incorrect one, maintaining an average accuracy of ~ 50%. Response times range from 0.5 s to 5.0 s in 0.5-second increments. Following each response or timeout, a fixation cross is shown for 0.5–5 s, followed by 0.5 s of visual feedback (“Right,” “Wrong,” or “Time out”).

Visual stimuli are presented using the Presentation Software (Neurobehavioral Systems, Inc.; Albany, CA, USA) and projected onto a translucent screen at the back of the scanner bore. Participants respond to arithmetic tasks using an MRI-compatible joystick, which triggers real-time visual feedback (“Right,” “Wrong,” or “Time out”). Prerecorded auditory feedback is presented through MRI-compatible headphones at four predetermined time points (i.e., after the first sets of nine trials) during each scan.

### Measurements

#### Psychological measures

All participants undergo the M.I.N.I. KID 6.0 [43], a structured clinical interview assessing DSM-IV and ICD-10 psychiatric disorders, to identify comorbid psychiatric disorders in the clinical groups or to confirm the absence of psychiatric diagnoses in healthy controls. Given evidence that childhood adversity can influence CORT responses to acute social stressors [44], retrospective assessment of maltreatment is conducted using the Childhood Trauma Questionnaire – Short Form (CTQ-SF) [45, 46]. The CTQ-SF is a validated 28-item measure covering sexual, emotional and physical abuse, and emotional and physical neglect. Items are rated on a five-point Likert scale (1 = “Never true”; 5 = “Very often true”). A recent meta-analysis supports its structural and construct validity [47].

Attachment anxiety and avoidance are measured with the German short version of the Experiences in Close Relationships Scale-Revised Child version (ECR-RC) [48, 49]. This self-report questionnaire includes 12 statements referring to participants’ relationship with their mother; six measuring attachment avoidance and six assessing attachment anxiety. Responses are rated on a seven-point Likert scale (1 = “strongly disagree”; 7 = “strongly agree”), with lower scores indicating higher attachment security. The ECR-RC has demonstrated excellent validity and reliability in youth samples [48]. Given that attachment patterns influence stress reactivity and OXT may modulate this relationship [50, 51], the ECR-RC was included to assess individual attachment differences in the context of OXT’s effects on stress.

Given the influence of handedness on lateralization [52], handedness is assessed using the Edinburgh Handedness Inventory (EHI; [53]). The inventory includes ten items assessing hand preference for specific tasks (e.g., drawing, eating with a spoon), from which a lateralization quotient (LQ) is calculated. Positive LQ values indicate right-handedness, while negative values reflect left-handedness.

Emotional reactivity is assessed using the Emotion Reactivity Scale (ERS), a 21-item self-report questionnaire for children and adolescents [54, 55]. The ERS assesses typical emotional response across interpersonal and situational contexts, including feelings of anger, sadness, and excitement. It includes three sub-dimensions: emotional sensitivity, intensity and persistence. Items are rated on a 5-point Likert scale (1 = “not at all like me”; 7 = “completely like me”), yielding a summed sore ranging from 0 to 84. The ERS has demonstrated excellent internal consistency (*α* = 0.94) and validity among adolescents [55].

Non-suicidal self-injury (NSSI) is assessed through self-report questions about participants’ NSSI history. Those reporting a positive history are asked about age of onset, most recent occurrence, and frequency of NSSI over their lifetime, past year, and past month.

Alexithymia is examined using the German version of the Perth Alexithymia Questionnaire, adapted for children and adolescents (PAQ-C) [56, 57]. This 24-item self-report measure is included to control for potential distortions in stress perception and reporting related to alexithymia. The PAQ-C assesses three subscales (difficulty identifying feelings, difficulty describing feelings, and externally oriented thinking) across both negative and positive emotions. Items are rated on a seven-point Likert scale (1 = “strongly disagree”; 7 = “strongly agree”), with higher scores indicating greater alexithymia. The PAQ has demonstrated strong psychometric properties across cultures [57–59].

Pubertal development is measured using the German version of the Pubertal Development Scale (PDS) [60, 61], a self-report questionnaire assessing physical maturation. Items assess developmental markers such as menarche, breast and pubic hair growth (females) and facial hair, voice changes, and pubic hair growth (males), relative to peers. Criterion validity and internal consistency measurements have demonstrated acceptable values [61]. Pubertal development influences CORT reactivity to acute psychosocial stress [62].

Social phobia is assessed using the German Screening for Social Phobia and Anxiety Inventory for Children (SPAIK) [63], based on DSM-IV criteria. The 26-item questionnaire covers psychological and physiological aspects of social phobia, with responses rated on a three-point Likert scale. The SPAIK has demonstrated strong cross-cultural validity and reliability in identifying social phobia in adolescents [64].

Anxiety symptoms are examined via the Beck Anxiety Inventory (BAI) [65, 66], a 21-item self-report scale measuring somatic and cognitive anxiety symptoms over the past seven days. The German version showed good psychometric properties, including strong convergent and divergent validity [67], and acceptable reliability in adolescent samples [68].

Depressive symptoms are assessed using the German version of the Beck Depression Inventory (BDI-II) [69, 70], a 21-item self-report scale for individuals aged 13 and older. Each item presents four statements of increasing severity, from which participants choose the one that applies most to them regarding the past two weeks. The German BDI-II shows excellent internal consistency (*α* ≥ 0.90), and reliability (>0.90), strong sensitivity to change, and good discriminative validity [71].

Dissociative states before and after neuroimaging are measured using the Dissociation-Tension Scale (DSS-4) [72], developed for repeated evaluations of state dissociation. It comprises four items (e.g., depersonalization, somatoform symptoms) rated on a 9-point Likert scale (0 = “not present”; 9 = “very strong”). The DSS-4 demonstrated good to excellent psychometric properties, including internal consistency, reliability, differential and convergent validity [72].

Participants’ current affective state during relaxation periods before and after the MRI is assessed using the German version of the Positive and Negative Affect Schedule (PANAS) [73, 74]. This 20-item self-report questionnaire comprises ten items each for positive (e.g., “enthusiastic,” “inspired”) and negative affect (e.g., “upset,” “ashamed”), rated on a 5-point Likert scale (1 = “not at all”; 5 = “extremely”). The German PANAS showed good psychometric properties [73].

Further, using Visual Analogue Scales (VAS) [75] (1 = “not at all”; 10 = “extremely”) participants are asked about how stressed, anxious, angry, ashamed, and guilty they feel at nine different time points throughout T2 (see Fig. 3). VAS are particularly suitable for assessing subjectively experienced stress, as they have been shown to be both reliable [76] and valid [77].

#### Saliva sampling

To analyse peripheral OXT, CORT and sAA levels, saliva samples are collected at eight time points using Salivettes^®^ (Sarstedt, Nümbrecht, Germany; item number 51.1534.500) following a standardized protocol [78]. Samples are stored at -20 °C until biochemical analysis. Salivary OXT will be analysed by radioimmunoassay (RIAgnosis, Sinzing, Germany). Salivary CORT and sAA will be quantified at the Department of Biopsychology, Technical University of Dresden, Germany.

#### Physiological consistency of measurement

All measurements are conducted on weekdays, with the experimental session (T2) scheduled exclusively in the afternoon to minimize circadian influences on hormone levels [79, 80]. For post-menarcheal females not using contraceptives, T2 is scheduled during the luteal phase (estimated based on self-reported cycle length, menstruation duration, and the date of last menstrual onset) to ensure comparability of CORT reactivity across sexes [81, 82]. Although no significant sex differences in OXT levels have been reported in adolescents [83], oral contraceptive use is assessed and statistically controlled, given evidence that such use blunts CORT reactivity [84]. Participants are instructed to refrain from eating and to only drink water two hours before and during T2, with compliance confirmed via self-report.

### MRI image acquisition

Imaging is conducted on a 3T Siemens Magnetom Prisma scanner (Siemens Healthineers, Erlangen, Germany) at the University of Regensburg using a 64-channel head/neck coil. rsFC is acquired before and after stress induction via MIST using a multi-band EPI sequence (470 volumes, 48 slices with isotropic voxel size of 2.5 × 2.5 × 2.5 mm³, 240 × 240 mm² FOV, TR = 1000 ms, TE = 30 ms, flig angle (FA) = 59° and multiband factor = 4 resulting in a total scan time of 8 min). Task-based fMRI uses a separate EPI sequence (233 volumes, 60 slices with isotropic voxel size of 2.5 × 2.5 × 2.5 mm³, 240 × 240 mm² FOV, TR = 2000 ms, TE = 30 ms and multiband factor = 4 resulting in a total scan time of 8 min). A gradient echo field mapping sequence (50 slices, isotropic voxel size of 2.0 × 2.0 × 2.0 mm³, 220 × 220 mm² FOV, TR = 645 ms, TE1 = 4.92 ms, TE2 = 7.38 ms resulting in a total scan time of 2 min and 27 s) is used to estimate B_0_ field inhomogeneities. Diffusion-weighted imaging is performed using a multi-band EPI sequence (72 slices with isotropic voxel size of 2.0 × 2.0 × 2.0 mm³, 212 × 212 mm² FOV, TR = 3900 ms, TE = 78.2 ms, diffusion weighting b = 2000 s/mm² applied along 136 noncollinear directions, including non–diffusion-weighted (b = 0) images acquired for reference and multiband factor = 2 resulting in a total scan time of 9 min and 33 s). High-resolution T1-weighted structural images are acquired using a Magnetization Prepared Rapid Acquisition Gradient Echo (MPRAGE) sequence (192 slices with isotropic voxel size of 0.9 × 0.9 × 0.9 mm³, 240 × 240 mm² FOV, TR = 2300 ms, TE = 2.32 ms, inversion time (TI) = 900 ms, FA = 8° resulting in a total scan time of 5 min and 21 s). T2-weighted structural images are acquired using a Fluid-Attenuated Inversion Recovery (FLAIR) sequence (26 slices with isotropic voxel size of 0.9 × 0.9 × 5.0 mm³, 230 × 230 mm² FOV, TR = 10000 ms, TE = 96 ms, TI = 2500 ms, FA = 150° resulting in a total scan time of 2 min and 42 s).

### MRI preprocessing

MRI data will be preprocessed using fMRIPrep [85, 86], a standardized pipeline that integrates best practices and ensures reproducibility. T1-weighted structural images will be skull-stripped and segmented into gray matter, white matter, and cerebrospinal fluid. Functional images will be corrected for head motion and slice-timing effects, and subsequently normalized to the MNI NIH Pediatric Asym template (Cohort 6, ages 13–18) [87, 88], which provides age-appropriate anatomical reference data for adolescent populations. Although fMRIPrep also produces derivatives in standard adult MNI space (MNI152NLin6Asym), all planned analyses will be conducted in the pediatric template space, with ROI (region-of-interest) masks coregistered accordingly.

### MRI and statistical analysis

In accordance with the preregistered clinical trial protocol (DRKS00032507), two primary outcome domains were originally defined: MRI measures and neuroendocrine responses. In the present manuscript, we focus primarily on rsFC within the fronto-limbic network and hormonal reactivity as the main outcomes. Some secondary outcomes specified in the DRKS protocol (e.g., psychological assessments) will be reported elsewhere. These deviations reflect updated hypotheses and advances in the field since the trial registration.

#### Primary outcomes

The first primary outcome is rsFC. We hypothesize that time (pre- vs. post-stress) × group (MDD, COM, HC) interactions will be observed in fronto-limbic connectivity.

Six a priori fronto-limbic ROIs will be examined: bilateral amygdala, hippocampus, ACC, insula, dorsolateral prefrontal cortex (dlPFC), and ventromedial prefrontal cortex (vmPFC), defined using the Harvard–Oxford cortical and subcortical atlases as implemented in the CONN toolbox [89]. This yields 15 possible pairwise connections. For each subject, mean time series will be extracted from each ROI, pairwise Pearson correlations will be computed, and coefficients will be Fisher’s z-transformed to normalize distributions. Group differences will be assessed using general linear models (GLMs) with time (pre- vs. post-stress) as within-subject factor and group (MDD, COM, HC) as between-subject factor. Sex assigned at birth, age, pubertal status (linear and quadratic terms), and medication status (yes/no) will be included as covariates. Additional psychiatric comorbidities (e.g., ADHD, OCD, PTSD, eating disorders) will be recorded but not included as routine covariates due to diagnostic heterogeneity and low subgroup frequencies; however, planned sensitivity analyses will be conducted excluding participants with additional comorbid diagnoses to assess robustness of findings. Due to the expected small number of left-handed participants, groups will not be matched on handedness; however, EHI scores will be considered as covariates in rsFC analyses. False Discovery Rate (FDR) correction across the 15 tested edges will be applied.

Exploratory analyses will extend this approach to a broader corticolimbic network comprising 25 ROIs previously identified as part of stress-sensitive circuitry [90–92]. For each ROI, the average time series will be extracted and rsFC estimated via Pearson correlations and Fisher’s z-transformation. The resulting z-values will serve as edge weights for subsequent network analyses. Multiple comparisons will be controlled using FDR correction across the 300 tested pairwise connections. In addition to the predefined ROI-to-ROI analyses, we will conduct exploratory, hypothesis-generating analyses to characterize stress-related changes in whole-brain connectivity. Specifically, each of the six a priori ROIs (bilateral amygdala, hippocampus, ACC, insula, dlPFC, vmPFC) will be used as seeds in seed-to-voxel analyses. rsFC will be estimated with all other voxels across the brain to identify potential alterations beyond the predefined corticolimbic circuitry. While these analyses are not restricted to predefined networks, particular attention will be paid to connectivity patterns involving large-scale systems, including the default mode, salience, and fronto-parietal networks, as defined by the Yeo 7-network parcellation and the Harvard–Oxford cortical and subcortical atlases. Multiple-comparison correction will be applied using FDR within each seed analysis. Primary analyses (fronto-limbic rsFC and hormonal reactivity) are treated as separate preregistered outcome domains and are each corrected using FDR procedures. Exploratory ROI-to-ROI and seed-to-voxel analyses are explicitly defined as hypothesis-generating and interpreted with caution, with FDR correction applied within each analytic family. This approach controls Type I error inflation while allowing exploratory analyses to inform future hypothesis development. This exploratory approach reflects the limited prior evidence in adolescent samples [92] and aims to generate hypotheses for future targeted studies.

The second primary outcome is hormonal reactivity. We hypothesize that stress exposure will elicit group differences in OXT, CORT and sAA. Raw hormonal values will be square-root transformed to reduce skewness [93]. Reactivity will be quantified using area under the curve (AUC) [94] with respect to increase (AUC_i_) and ground (AUC_g_), as well as delta scores reflecting change from baseline to peak, peak to recovery, and recovery magnitude [95–97]. Repeated-measures ANOVAs will examine the interaction between time (baseline, peak, recovery; within-subject factor) and group (between-subject factor), controlling for sex, age, and pubertal status. False Discovery Rate (FDR) correction will be applied across hormonal outcomes and indices. Subjective stress ratings assessed with VAS will be analysed in parallel, using repeated-measures ANOVAs with the same model structure.

#### Secondary outcomes

Structural MRI measures will be derived from T1- and T2-weighted images to calculate cortical thickness, surface area, and subcortical volumes. Group comparisons will be conducted using ANCOVAs, controlling for sex, age, and pubertal status, with FDR correction applied across regions.

DTI data will be preprocessed with QSIPrep [98] for denoising, motion and distortion correction, followed by whole-brain tractography and structural connectome reconstruction as outlined in recent protocols [99]. Cortical parcellation will be based on the Brainnetome atlas, and structural connectivity matrices will be derived from streamline counts and weighted by fractional anisotropy. Graph-theoretical measures (e.g., node strength) will be extracted using the Brain Connectivity Toolbox. Between-group differences will be tested using ANCOVAs including sex, age or pubertal status, and medication status (yes/no) as covariates.

Task-based fMRI will be analysed using GLMs to assess BOLD activation during the stress induction task. At the first level, subject-specific GLMs will be estimated, and at the second level, group differences will be examined using ANOVAs. The primary contrast will compare stress versus baseline, with sex, age or pubertal status, and medication status (yes/no) included as covariates.

Exploratory analyses will include depressive and anxiety symptomatology scores as covariates in rsFC, sMRI, and hormonal models to test whether associations remain robust after accounting for clinical variation. Sex assigned at birth will be recorded, and exploratory analyses will examine group × sex interactions. When appropriate, non-parametric tests (e.g., Mann–Whitney U-tests) will be applied in case of non-normal distributions. Relationships between VAS ratings and hormone levels (OXT, CORT, sAA) will be assessed using bivariate correlations. Multiple regression models will also be applied to assess associations between hormonal systems. In the first model, CORT increase will be the dependent variable, with OXT increase, baseline CORT, and group as predictors. In the second model, CORT recovery will be the dependent variable, with OXT and CORT increase as predictors.

Across all analyses, effect sizes will be reported using Cohen’s d (0.2 = small, 0.5 = medium, 0.8 = large). Unless otherwise adjusted for multiple comparisons, the alpha threshold will be set at 0.05. Data will be analysed using the CONN Toolbox for neuroimaging, and IBM SPSS Statistics (Version 29.0, IBM Corp.) and jamovi (Version 2.6.22, The jamovi project, 2024) for behavioural and hormonal analyses.

### Expense allowance

Adolescents participating in our study receive a gift voucher of €50 as an expense allowance for participation.

### Adverse event reporting and harms

No adverse events are expected from study participation, and medically necessary treatments will not be delayed or altered. In the event of significant symptom worsening, therapeutic support will be offered. Participation is voluntary, and both adolescents and their legal guardians may withdraw at any time without providing a reason.

## Discussion

### Gains in knowledge

This study aims to deepen our understanding of how psychosocial stress modulates fronto-limbic connectivity and neuroendocrine responses in adolescents with MDD, with and without comorbid anxiety. While previous research has largely focused on adults, this study targets a crucial developmental window by examining stress-related changes in rsFC and peripheral markers of the HPA axis and OXT system in youth. We anticipate that adolescents with MDD will exhibit reduced rsFC between the amygdala and prefrontal regions at baseline, with more pronounced hypoconnectivity in those with comorbid anxiety. If confirmed, these patterns may reflect impaired top-down emotional regulation and underscore the need for interventions that enhance prefrontal control mechanisms.

By assessing stress-induced changes in connectivity and hormonal reactivity, this study seeks to identify potential biomarkers of stress adaptation. For instance, if blunted CORT and OXT responses are linked to altered rsFC patterns in clinical groups, this may point to specific neuroendocrine dysregulations in affective circuit modulation. Such findings could inform the refinement of neuromodulation techniques such as Transcranial Magnetic Stimulation or neurofeedback [21, 100], by targeting brain regions or connectivity profiles associated with impaired stress responses. Moreover, identifying distinct patterns between adolescents with depression alone and those with comorbid anxiety may support the development of stratified treatment approaches based on individual neurobiological profiles rather than diagnostic categories alone.

Although the cross-sectional design limits causal inference, consistent associations between rsFC, endocrine reactivity, and symptom profiles may inform future longitudinal research and early intervention strategies. Integrating neuroimaging and endocrine data holds promise for refining mechanistic models of adolescent depression and anxiety, ultimately supporting the development of more personalized, biologically informed mental health care.

### Strengths and limitations

A key strength of this study is its multimodal design, integrating fMRI, DTI, and endocrine biomarker analysis to provide a comprehensive view of brain structure and function in response to stress. By including adolescents with comorbid anxiety disorders, a group often overlooked in neuroimaging studies, the project enhances ecological validity and clinical relevance. The sample is well-characterized: clinical diagnoses are made by experts and confirmed with structured interviews, while healthy controls are screened for psychiatric conditions. Further, potential confounders like age, school type, pubertal status, and handedness are controlled to ensure comparability across groups.

Several limitations also must be acknowledged. The clinical sample is heterogeneous in terms of comorbidities, medication use, and therapy status, which may introduce variability in stress responses. Within the comorbid anxiety disorder group, diagnostic heterogeneity is also present, as anxiety disorders comprise distinct conditions (e.g., panic disorder, generalized anxiety disorder) that may differ in genetic contributions and neural activation patterns. Due to recruitment and sample size constraints, subgroup analyses by anxiety disorder type will not be performed. While this approach reflects clinical reality, it may also introduce variability unrelated to our primary research question. Such diversity reflects real-world populations but could obscure more subtle effects. The relatively large sample size (*N* = 90) helps offset this limitation by increasing statistical power. Ongoing therapy in some participants may influence stress responses through learned coping mechanisms; however, given the short timeframe, these effects are unlikely to significantly alter rsFC findings. Additionally, the cross-sectional design forbids causal inferences, underscoring the need for longitudinal research to track changes in connectivity and endocrine function over time or in response to treatment. Finally, while this study focuses on neurobiological and hormonal mechanisms, it remains unclear whether the most effective interventions should target these systems, psychological processes, or both—an important question for future research.

Despite these limitations, this study offers significant contributions to understanding adolescent stress reactivity in depression and anxiety. It lays important groundwork for future research aimed at developing targeted, biologically informed interventions that account for the complex interplay between brain, behavior, and hormonal systems.

## Data Availability

Data sharing is not applicable to this article as no datasets were generated or analysed during the current study. Data generated during the study will be made available upon reasonable request in the subsequent results publication.

## Abbreviations

ANOVA: Analysis of Variance
ACC: Anterior Cingulate Cortex
AUC: Area under the Curve
AUC_i_: Area under the Curve with respect to increase
AUC_g_: Area under the Curve with respect to ground
BAI: Beck Anxiety Inventory
BDI–II: Beck Depression Inventory–II
COM: Group with comorbid depressive and anxiety disorder
CORT: Cortisol
CTQ-SF: Childhood Trauma Questionnaire–Short Form
dlPFC: Dorsolateral Prefrontal Cortex
DSM–IV: Diagnostic and Statistical Manual of Mental Disorders, 4th edition
DSS–4: Dissociation–Tension Scale
DTI: Diffusion Tensor Imaging
ECR–RC: Experiences in Close Relationships Scale–Revised Child version
EHI: Edinburgh Handedness Inventory
EPI: Echo planar imaging
ERS: Emotion Reactivity Scale
FA: Flip angle
FDR: False Discovery Rate
FLAIR: Fluid–Attenuated Inversion Recovery
fMRI: Functional Magnetic Resonance Imaging
FOV: Field of view
GLM: General Linear Model
HC: Healthy control group
HPA: Hypothalamic–pituitary–adrenal
ICD–10: International Classification of Diseases, 10th revision
LQ: Lateralization Quotient
MDD: (Group with) Major Depressive Disorder
M.I.N.I.–KID: Mini–International Neuropsychiatric Interview for Children and Adolescents
MIST: Montreal Imaging Stress Task
MPRAGE: Magnetization Prepared Rapid Acquisition Gradient Echo
MRI: Magnetic Resonance Imaging
OXT: Oxytocin
PANAS: Positive and Negative Affect Schedule
PAQ: C–Perth Alexithymia Questionnaire–Child version
PDS: Pubertal Development Scale
rmANOVA: Repeated measures Analysis of Variance
rsFC: Resting–state functional connectivity
ROI: Region of interest
sAA: Salivary alpha–amylase
SPAIK: Screening for Social Phobia and Anxiety Inventory for Children
T1/T2: Time points 1 and 2
TE: Echo time
TI: Inversion time
TR: Repetition time
VAS: Visual Analogue Scale
vmPFC: Ventromedial Prefrontal Cortex
